# Organization and regulation of intracellular plasma membrane-connected HIV-1 assembly compartments in macrophages

**DOI:** 10.1186/1741-7007-11-89

**Published:** 2013-08-02

**Authors:** Petra Mlcochova, Annegret Pelchen-Matthews, Mark Marsh

**Affiliations:** 1 Medical Research Council Laboratory for Molecular Cell Biology, University College London, Gower Street, London WC1E 6BT, UK

**Keywords:** Actin cytoskeleton, Compartment, HIV-1, IPMC, MDM

## Abstract

**Background:**

In HIV-1-infected human monocyte-derived macrophages (MDMs), virus particles assemble primarily on intracellularly sequestered plasma membrane domains termed intracellular plasma membrane-connected compartments (IPMCs). Despite their clear role in virus formation, little is known of the organization, composition, dynamics or function of these compartments.

**Results:**

We have used amphipathic membrane dyes to reveal the complex three-dimensional structure of IPMCs in whole MDMs and to visualize connections between IPMCs and the cell surface. The observation of similar IPMC structures in both infected and uninfected cells indicates that these compartments are not induced by virus infection, but are present constitutively in MDMs. By expressing a phospholipase Cδ pleckstrin homology domain linked to green fluorescent protein, we demonstrate that IPMCs contain phosphatidylinositol 4,5-bisphosphate. Live cell imaging of cells expressing this probe shows that IPMCs are dynamic, but relatively stable, sub-domains of the plasma membrane. As recent electron microscopy studies indicated that portions of IPMCs are coated with β2 integrin-containing focal adhesion-like complexes linked to actin, we investigated whether the actin cytoskeleton is required for the organization of IPMCs. In MDMs treated with the actin polymerization inhibitor latrunculin, the normally compact IPMCs dispersed into smaller structures that remained connected to the plasma membrane. Moreover, latrunculin enhanced the release of preformed, mature HIV-1 particles from infected MDMs.

**Conclusions:**

IPMCs are constitutive features of MDMs that are continuous with the plasma membrane and are used as unique sites for the assembly of new virions following infection by HIV-1. A functionally intact actin cytoskeleton is required to maintain the organization of the IPMCs and, in HIV-1-infected cells, perturbation of the actin cytoskeleton influences both the organization of the compartment and the release of sequestered virus.

## Background

CD4^+^ T-cells and macrophages are the major targets for HIV-1 infection *in vivo*[1]. Unlike T-cells, macrophages can survive for long periods of time following infection, potentially serving as reservoirs of infectious virus [2] that may facilitate the spread of virus to other target cells and to the brain [3-5]. Previous work has shown that in monocyte-derived macrophages (MDMs) differentiated in culture and infected with HIV-1, new virus particles assemble on intracellular membranes variously suggested to be derived from the Golgi apparatus [6] or endosome-associated compartments [7-9]. However, recent studies have shown that HIV assembly in MDMs takes place on membranes that are connected to the plasma membrane [10-16]. In HIV-infected MDMs, these intracellular plasma membrane-connected compartments (IPMCs) appear to be the primary sites of virus assembly, as budding profiles, immature and mature virus particles can be seen within the IPMC structures, but are only rarely observed at the cell surface [10,14,17]. Electron microscopy (EM) has revealed IPMCs to be complex networks of membranes comprising tubules and vacuoles that can be connected to the cell surface via narrow channels [10,11,13-15]. Although the function of IPMCs in MDMs is currently unclear, their presence in uninfected cells [11,14,15] indicates that they are not solely involved in virus replication but are likely to have other function(s) in macrophages.

Although well described morphologically, mainly in fixed samples, little is known about the formation, function or dynamics of IPMCs. Here, we used the membrane dyes FM 4–64 and CellMask to investigate IPMCs and to further understand their links to the cell surface. In addition, using a green fluorescent protein (GFP)-tagged pleckstrin homology (PH) domain from phospholipase Cδ (PH-GFP), a probe that binds specifically to the lipid phosphatidylinositol 4,5-bisphosphate (PI(4,5)P_2_), we could label IPMCs and monitor their behavior in living MDMs. One characteristic feature of IPMCs is the presence of areas of electron-dense coat material on the cytoplasmic side of the limiting membrane, close to meshworks of cytoskeletal filaments [11,14]. These coats are related to focal adhesion plaques, containing the integrins αM/αX β2 (CD11b,c/CD18), together with the focal adhesion proteins talin, paxillin and vinculin that link to the actin cytoskeleton [14]. We therefore also investigated the role of the actin cytoskeleton in the organization of IPMCs and the release of mature, IPMC-sequestered HIV-1.

## Results

### Imaging of IPMCs with lipophilic membrane dyes

For the most part, IPMCs have been studied with regard to their role in HIV-1 replication in macrophages. However, previous work has suggested that IPMCs are also present in uninfected MDMs and can be identified by immunostaining for membrane proteins such as CD9, CD81 or the hyaluronic acid receptor CD44 [11,14,15]. As IPMCs are thought to be continuous with the plasma membrane, we investigated the ability of lipophilic dyes added to the cell media to label these compartments.

**Figure 1:**
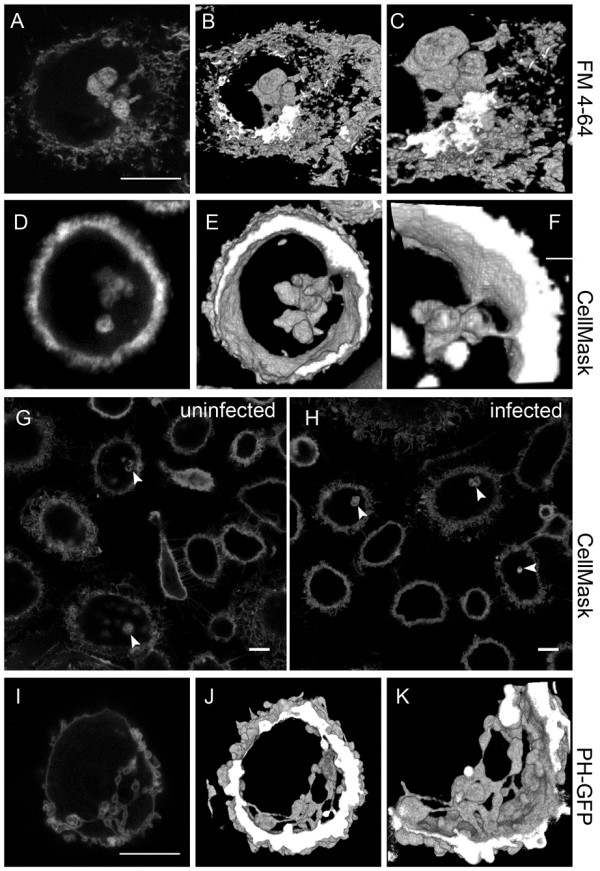
**Visualization of IPMCs in MDMs. (A**-**C)** Uninfected MDMs were labeled with 5 μg/ml FM 4-64FX, fixed and analyzed by confocal microscopy. **(A)** Shows a single optical section, **(B)** a three-dimensional reconstruction assembled from 131 optical z-slices (step size of 0.04 μm), and **(C)** a detail of the IPMC shown in B. See also Additional file 1. **(D**-**F)** Live cell imaging of uninfected MDMs labeled with CellMask; **(D)** shows a single section, **(E)** a three-dimensional reconstruction assembled from 165 optical z*-*slices (step size of 0.1 μm)*,* and **(F)** a detail of the IPMC. See also Additional file 3. Cells were imaged using an UltraVIEW Vox spinning disc confocal system (PerkinElmer, Cambridge, UK) fitted on a Nikon ECLIPSE Ti microscope equipped with a temperature and CO_2_-controllable environment chamber. **(G**,**H)** Uninfected MDMs **(G)** or MDMs infected with HIV-1 for 7 days **(H)** were labeled with the membrane-impermeable dye CellMask and fixed. The images show single confocal sections. Arrowheads mark CellMask-stained IPMCs. **(I**-**K)** Uninfected MDMs were nucleofected with an expression plasmid encoding PH-GFP and 24 hours later were fixed and imaged by confocal microscopy; **(I)** shows a single section, **(J)** a three-dimensional reconstruction assembled from 230 optical z-slices (step size of 0.04 μm)*,* and **(K)** a detail of the IPMC. See also Additional file 4. The confocal images were acquired with a Leica TCS SPE confocal microscope, 63× oil objective (NA 1.3) and Leica LAS Software, and processed using Adobe Photoshop, ImageJ and Fiji. All experiments were repeated with cells from at least three different donors. All scale bars = 10 μm. IPMC: intracellular plasma membrane-connected compartments; PH-GFP: Phospholipase Cδ pleckstrin homology domain linked to GFP.

**Figure 2:**
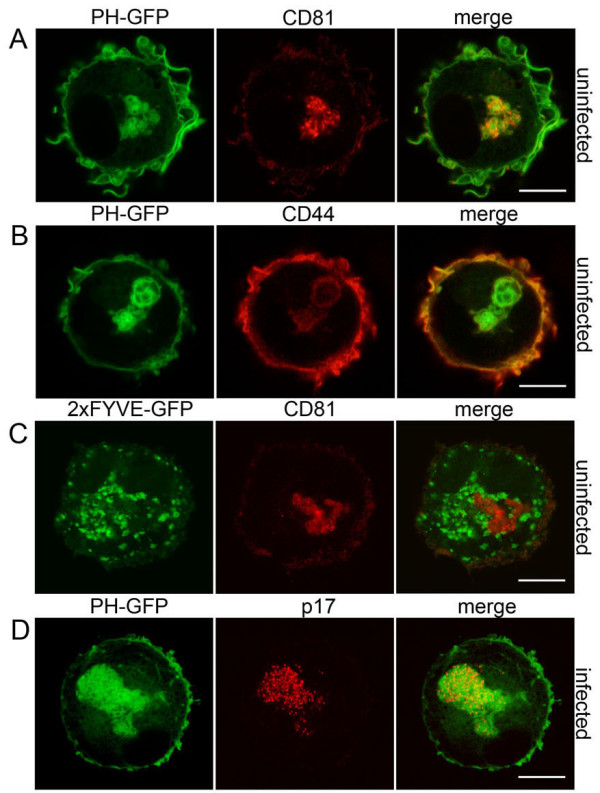
**Localization of PI*(*4*,*5)P**_**2 **_**and phosphatidylinositol 3-phosphate in MDMs.** MDMs were nucleofected to express PH-GFP, to localize **(A**,**B)** PI(4,5)P_2_ or **(C)** a double-FYVE domain construct fused to GFP (2xFYVE-GFP), a probe for phosphatidylinositol 3-phosphate. After 24 hours the cells were fixed and co-stained for CD81 or CD44 as indicated. **(D)** Seven-day-old MDMs were infected with HIV-1_BaL_ and after a further 7 days were nucleofected to express PH-GFP, incubated for 24 hours, then fixed and stained for the viral matrix protein p17. Images show single confocal sections, and were acquired with a Leica SPE confocal microscope as in Figure 1. All experiments were repeated on cells from at least three different donors. Scale bars = 10 μm. PI(4,5)P2: Phosphatidylinositol 4,5-bisphosphate; PH-GFP: Phospholipase Cδ pleckstrin homology domain linked to GFP.

Because IPMCs in MDMs have been shown to develop with time in cell culture [14], we used 14-day-old MDMs for all of the studies described here. Uninfected MDMs were incubated with FM 4-64FX, a formaldehyde/glutaraldehyde-fixable analog of FM 4–64, a lipophilic dye that fluoresces intensely on binding to the outer leaflet of lipid bilayers (Figure 1A-C). Labeling was carried out on ice to prevent endocytosis, through which the dye might access intracellular membranes by vesicular carriers [18]. Subsequently, the cells were fixed and imaged. This allowed rapid and specific labeling of the cell surface plasma membrane. In addition, FM 4–64 labeling was seen in prominent intracellular structures reminiscent of CD9-, CD81- or CD44-labeled IPMCs (Figure 1A-C). In three-dimensional reconstructions of FM 4-64FX-labeled cells from optical sections, the IPMCs appeared as clusters of interconnected vacuoles with occasional direct contacts to the cell surface (Figure 1A-C and Additional file 1). Because the cells were stained on ice in the absence of endocytosis, and we saw no labeled vesicles resembling endocytic organelles (compare with Figure 2C), we assume that these connections allowed the dye to access the IPMCs. To confirm that IPMCs labeled with FM 4–64 correspond to the compartments where HIV assembles, we expressed the HIV structural protein Gag linked to GFP (Gag-GFP), which is also targeted to CD81- and CD44-positive IPMCs in MDMs (AP-M, unpublished data), and stained these cells with FM 4-64FX. In transfected MDMs, both Gag-GFP and FM 4–64 co-localized in IPMCs (Figure S1A in Additional file 2).

In an alternative approach, we used CellMask Plasma Membrane Stain, a negatively charged amphipathic molecule reported to undergo only slow internalization, to label MDMs. Cells were incubated with the dye for 5 minutes at 37°C or 30 minutes at 4°C, fixed and analyzed by confocal microscopy. As with FM 4-64FX, CellMask labeled the cell surface as well as surface-connected IPMCs (Figure 1D-F). Because the labeling showed the same pattern at both temperatures (Figure S1B in Additional file 2), we labeled cells for 5 minutes at 37°C for all further experiments with CellMask. The slow internalization of this dye also allowed us to observe IPMCs in living cells by spinning disc confocal microscopy for up to 20 minutes before uptake of the dye into endosomes became apparent. This allowed us to image whole cells and IPMCs in three dimensions while avoiding possible changes associated with fixation and without simultaneous labeling of endosomes and/or lysosomes (Figure 1D-F and Additional file 3). Together these studies indicated that IPMCs in uninfected MDMs can contain several interconnected vacuole-like structures with connections to the cell surface, though the numbers of connections varied for individual cells. For example, in 11 different three-dimensional reconstructions of CellMask-stained MDMs, three IPMCs had a single connection to the cell surface, while eight had two or more. Similarly, in five FM 4-64-labeled MDMs, four IPMCs showed two or more connections to the cell surface.

We also used CellMask to label 14-day-old uninfected MDMs and equivalent cells (from the same donor) that had been infected with HIV-1 for 7 days (Figure 1G,H). Most of the cells showed labeling of intracellular compartments, although there was significant cell-to-cell variability in the size and morphology of the IPMCs (several examples are documented in Figure S1B in Additional file 2). To determine whether HIV infection induces the *de novo* formation of IPMCs, we inspected single optical sections such as those shown in Figure 1G, H and counted the numbers of cells with prominent CellMask-labeled IPMCs (that is, structures >0.9 μm, where 0.9 μm was set as the lower limit for analysis) in the uninfected and HIV-infected MDMs. In this analysis, 28 ± 5.3% of the uninfected and 31 ± 2.4% of the cells in the infected MDM cultures contained IPMCs (200 cells counted), indicating that HIV infection does not itself induce the formation of IPMCs. As CellMask staining does not survive detergent extraction, co-staining for virus was not possible. However, independent labeling of MDMs for the HIV-1 matrix protein (p17) showed that approximately 22 ± 5% of the cells were producing mature virus (200 cells counted), with most viral staining located in the IPMCs. Together, these studies show that IPMCs, similar to the previously described HIV assembly compartments, are present in uninfected as well as HIV-1 infected cells, connected to the cell surface, and exhibit high cell-to-cell morphological variability.

### IPMCs contain PI(4,5)P_2_

The phosphoinositide PI(4,5)P_2_, which is located primarily in the cytoplasmic leaflet of the plasma membrane [19], is essential for targeting of HIV-1 Gag to the plasma membrane prior to virus assembly [20,21]. Because HIV assembly in MDMs takes place in IPMCs, we asked whether PI(4,5)P_2_ is also present in these compartments. Uninfected MDMs were nucleofected with an expression construct encoding the PH domain of phospholipase Cδ fused to GFP (PH-GFP), which specifically binds PI(4,5)P_2_. After 24 hours, the cells were fixed and stained for CD81 or CD44. PH-GFP was detected at the cell surface plasma membrane and also in IPMCs (Figure 1I), where it co-localized with CD81 and CD44 (Figure 2A,B). As with the membrane-impermeable dyes, PH-GFP outlined the membranes of IPMCs, revealing complex interconnected membrane structures with larger, vacuole-like components linked by fine membrane tubules or channels that also occasionally connected them to the cell surface (Figure 1I-K and Additional file 4). Furthermore, the compartments outlined by PH-GFP were morphologically variable, even between cells from the same donor; most of the MDMs contained one single complex IPMC but others showed more dispersed smaller IPMCs (Figure S1C in Additional file 2), and many of the IPMCs had more than one connection to the cell surface. By contrast, in MDMs nucleofected to express a double-FYVE domain construct fused to GFP (2×FYVE-GFP), which binds specifically and with high affinity to phosphatidylinositol 3-phosphate (a lipid associated primarily with early endosomes and the intralumenal vesicles of multivesicular endosomes [22]), vesicles were seen with a distribution that was clearly distinct from the structures stained with PH-GFP, CellMask or FM 4–64 dye and that did not overlap with the CD81-labeled IPMCs (Figure 2C).

To confirm the presence of PI(4,5)P_2_ in the IPMCs, we labeled MDMs with the mouse monoclonal anti-PI(4,5)P_2_ antibody 2C11 [23,24] using two different labeling protocols (see Methods). Labeled IPMCs were rarely seen on saponin-permeabilized cells, but after permeabilization with Triton X-100, most IPMCs were strongly stained by the PI(4,5)P_2_-specific antibody (Additional file 5).

We also infected 7-day-old MDMs with HIV-1 and 6 days later, nucleofected them with the PH-GFP expression construct. After 24 hours the cells were fixed and labeled for the viral matrix protein p17. In the infected cells expressing PH-GFP, both PH-GFP and p17 clearly co-localized in IPMCs (Figure 2D and Figure S1C in Additional file 2). However, MDMs containing both PH-GFP and p17 labeling were rare, suggesting that HIV-infected cells may be more difficult to transfect or, possibly, that the PH-GFP might compete with HIV-1 Gag for PI(4,5)P_2_ binding, though this has not been tested directly.

The presence of PI(4,5)P_2_ on IPMCs, together with our previous data showing the plasma membrane markers CD44 and CD81 as well as the endocytic clathrin adaptor complex AP2 on IPMCs [14], further supports the notion that IPMCs are intracellularly sequestered domains of the plasma membrane present in uninfected and HIV-infected MDMs.

### Live cell imaging of IPMCs

**Figure 3:**
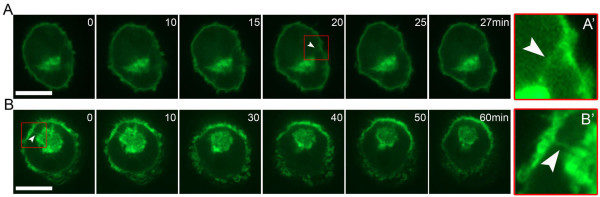
**Live cell imaging of IPMCs.** MDMs were nucleofected to express PH-GFP and imaged after 24 hours using an UltraVIEW Vox spinning disc confocal system, as described in Figure 1 (see Additional files 6 and 7). Images were recorded at 6 frames/minute with a 60× Nikon oil immersion objective (NA 1.4) using Volocity 5.3.2 (Perkin Elmer) and the Perfect Focus System, and analyzed with Volocity, Adobe Photoshop, ImageJ and Fiji software. The white arrowheads in **A** and **B** show connections to the cell surface. The images **A**’, **B**’ at the right show enlargements of the areas marked by the red boxes. All experiments were repeated on cells from at least three different donors. Scale bars = 20 μm.

Expression of PH-GFP allowed us to study the behavior of IPMCs in living MDMs by spinning disc confocal microscopy. All analyzed PH-GFP-labeled IPMCs appeared stable during the recording periods (usually 1 hour; Figure 3A,B; Additional files 6 and 7), though the structures were dynamic, and, in some cases, connections to the cell surface were observed (Figure 3 arrowheads).

**Figure 4:**
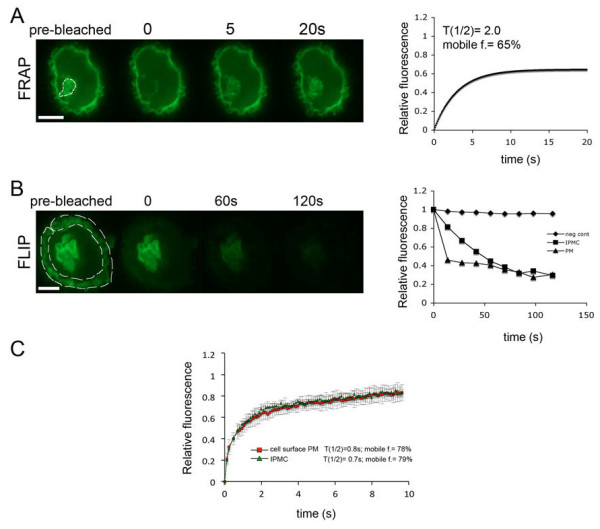
**FRAP and FLIP analysis of MDMs expressing PH-GFP.** MDMs were nucleofected to express PH-GFP and FRAP or FLIP analysis was performed 24 hours later using an UltraVIEW Vox spinning disc confocal system. Images were recorded at 6 frames/minute with a 100× Nikon oil immersion objective (NA 1.4) with Volocity 5.3.2 (Perkin Elmer). **(A)** FRAP: The PH-GFP-labeled IPMC (white selection) was bleached with a 20 millisecond laser pulse (488-nm laser at 50% intensity), and recovery of fluorescence was measured for 20 seconds by collecting frames at maximum speed. Data are shown in the graph on the right. **(B)** FLIP: The cell surface plasma membrane (white selection) was repeatedly bleached with 10 × 20 millisecond pulses of the 488 nm laser at 50% intensity using the UltraVIEW PK device. After each bleaching pulse, the fluorescence was monitored at various areas of interest for 10 seconds at maximum speed. The bleach and recovery cycle was repeated 10 times. The graph on the right shows the levels of fluorescence at the cell surface plasma membrane (PM, the photobleached area) or at the IPMC, compared to the plasma membrane of a different cell away from the bleached region (negative control). All experiments were repeated on cells from at least two donors. **(C)** Comparison of FRAP at the cell surface over IPMC membranes. Selected 2 × 2 μm^2^ areas at the cell surface or over IPMCs were photobleached, and FRAP was measured. The graph shows the average of 10 measurements in different cells from the same donor. Scale bars = 10 μm.

To investigate the dynamic behavior of IPMCs, we used fluorescence recovery after photobleaching (FRAP) and fluorescence loss in photobleaching (FLIP) techniques on MDMs expressing PH-GFP (Figure 4). For the FRAP experiments, the entire IPMC within a cell was photobleached and the recovery of fluorescence was monitored. Fluorescence recovered quickly in the bleached areas, with a mean half time of recovery of 2.7 seconds (range 1.2 seconds to 3.4 seconds; 13 cells recorded) and mobile fraction of 70% (Figure 4A). Similar recovery half times and mobile fractions were observed in FRAP studies when we compared the cell surface plasma membranes and IPMC membranes (Figure 4C). For the FLIP experiments, the cell surface membrane was repeatedly bleached with 20 millisecond pulses of laser illumination and the decay of the fluorescence signal of the corresponding IPMC was measured by acquiring an image of the sample after each photobleaching pulse. There was a clear decrease in the fluorescence signal in the IPMCs as the cell surface plasma membrane was bleached (Figure 4B). Similarly, in experiments where the IPMC was bleached within the cells, we observed a loss in the cell surface plasma membrane fluorescence intensity (data not shown). Together, the FRAP and FLIP data support the notion that IPMCs can exchange PH-GFP through lateral diffusion or cytosol-membrane exchange or a combination of both. These experiments show, for the first time, the behavior of IPMCs in uninfected, living MDMs and demonstrate that they are stable but dynamic structures.

### The actin cytoskeleton maintains the integrity of the IPMC

**Figure 5:**
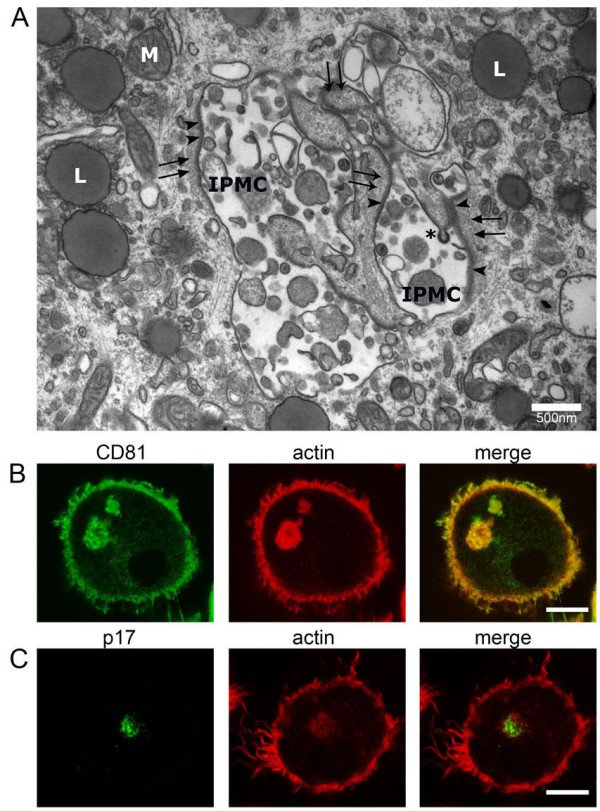
**Association of actin with IPMCs. (A)** HIV-infected MDMs were embedded in Epon, sectioned and analyzed by transmission EM, as previously described [14]. HIV particles are seen in a complex IPMC. Some of the IPMC membranes are covered with a coat of electron-dense focal adhesion proteins (arrowheads) and a layer of fine filaments (black arrows). L, lipid droplets; M, mitochondrion; the asterisk marks a budding HIV particle. Epon EM was conducted on MDMs from two donors. Scale bar = 500 nm. **(B)** Uninfected MDMs were stained for CD81 and actin (Alexa Fluor 594-conjugated phalloidin). **(C)** HIV-1-infected MDMs were stained for the matrix protein p17 and actin. The images show single optical sections acquired with a Leica SPE confocal microscope as above. Scale bars = 10 μm.

Our recent EM analysis of HIV-infected MDMs revealed a meshwork of actin filaments adjacent to β2-integrin-containing electron-dense membrane coats on HIV-containing IPMCs [14]. Similar filament meshworks can be seen in Figure 5A (arrows). We confirmed the presence of actin near the IPMCs by staining with Alexa Fluor 594-phalloidin and co-labeling for CD81 on uninfected MDMs (Figure 5B), or with anti-p17, identifying mature virions, on HIV-infected cells (Figure 5C).

**Figure 6:**
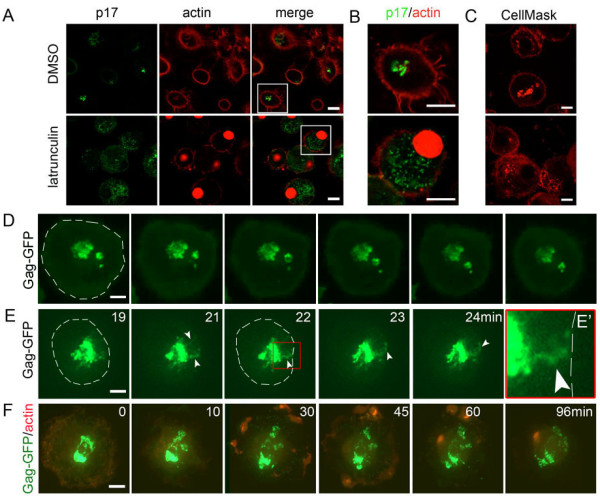
**Actin assembly is required for the integrity of the IPMCs. (A-C)** HIV-infected MDMs were treated with medium containing dimethyl sulfoxide (control) or 2 μM latrunculin A for 2 hours. **(A)** Cells were stained with an anti-p17 antibody that only recognizes mature virus particles and Alexa Fluor 594-conjugated phalloidin to label actin. **(B)** Enlargements of the marked areas in panel A. The images show single optical sections acquired with a Leica SPE confocal microscope as above. **(C)** HIV-infected macrophages were labeled with CellMask for 5 minutes at 37°C, fixed and imaged with an UltraVIEW Vox spinning disc confocal system as described in Figure 3 (see Additional file 11). **(D**,**E)** MDMs were nucleofected to express Gag-GFP and imaged after 24 hours using an UltraVIEW Vox spinning disc confocal system as described above (see Additional files 14 and 15). Images were recorded at 6 frames/minute with a 60× Nikon oil immersion objective (NA 1.4) using Volocity 5.3.2 (Perkin Elmer) and analyzed with Volocity, Adobe Photoshop, ImageJ and Fiji software. The cell periphery is indicated with the dashed white line. The white arrowheads in **(E)** show Gag-GFP in thin channels between the IPMC and the cell surface. The image E’ shows an enlargement of the area marked by the red box. **(F)** MDMs, co-transfected to express Gag-GFP and LifeAct-Ruby for 24 hours, were treated with 2 μM latrunculin A and imaged for 1.5 hours with the spinning disc confocal system (see also Additional file 16). All experiments were repeated with cells from at least two different donors. All scale bars = 10 μm.

To investigate whether actin plays a role in maintaining the integrity and organization of the IPMCs, HIV-infected MDMs were treated with the actin polymerization inhibitor latrunculin. Immunofluorescence staining showed that, in control cells, p17-containing mature HIV was located in large IPMCs and associated with actin (Figures 5 and 6A,B). After 2 hours of latrunculin treatment, actin was largely depolymerized and accumulated in the nuclei (Additional file 8), as previously described for latrunculin-treated mast cells [25]. At the same time, the staining for intracellular virus (p17) was dispersed throughout the cells (Figure 6A,B). A similar effect was observed when actin was depolymerized by cytochalasin E or D (Additional file 9). To quantify this effect, we counted the numbers of MDMs with compact, dispersed, or mixed (compact and dispersed) IPMCs in control MDMs or in cells treated with latrunculin, cytochalasin E or cytochalasin D. Perturbation of the actin cytoskeleton led to a statistically significant decrease in the number of cells with compact IPMCs (*P* <0.01) and a concomitant increase in the number of cells with dispersed compartments (Figure S4B,C in Additional file 9). Immuno-EM analysis of latrunculin-treated, HIV-infected MDMs demonstrated that HIV and immature budding particles were still found in intracellular compartments, although the IPMCs appeared more dispersed throughout the cells (Additional file 10).

To study the structure of the IPMCs in latrunculin–treated cells more directly, infected MDMs were labeled with the CellMask dye. This again revealed that latrunculin treatment caused the dispersal of the usually compact IPMCs into smaller structures (Additional file 11). CellMask labeling demonstrated that these smaller, more scattered IPMC structures were accessible from the cell surface; indeed in three-dimensional reconstructions, some connections to the cell surface were apparent (Additional file 11). Likewise, when MDMs were transfected with PH-GFP for 24 hours and then treated with latrunculin, the labeled compact IPMC dispersed to a network of membranes with several connections to the cell surface (Additional files 12 and 13). These observations suggest that actin depolymerization causes the usually tightly packed IPMC membranes to open up, giving rise to a more reticular network of membranes throughout the cell.

We also studied the behavior of IPMCs in the presence of HIV-1 Gag-GFP and the effect of disrupting the actin cytoskeleton by live cell imaging. MDMs were nucleofected to express Gag-GFP and monitored by spinning disc confocal microscopy. Accumulations of Gag-GFP, presumably virus-like particles and/or Gag-GFP protein clusters, were seen in intracellular structures resembling the IPMCs that appeared stable for over one hour without any significant rearrangements (Figure 6D and Additional file 14). Occasionally Gag-GFP was also seen in thin channels emanating from the IPMCs (Figure 6E and Additional file 15), perhaps equivalent to the virion-channeling tube-like structures described by Bennett *et al*. [10].

In experiments where MDMs were nucleofected to express Gag-GFP together with LifeAct-Ruby to label actin [26] and monitored for 1.5 hours after addition of latrunculin, the compact IPMCs initially seen in most cells (Figure 6D,E and Figure 6F, 0 minute time point) became more dispersed after 20 minutes of latrunculin treatment. The Gag-GFP appeared more mobile in latrunculin-treated cells (compare Additional files 14 and 15 with Additional file 16), and, over 60 minutes, the IPMCs in latrunculin-treated cells scattered into smaller structures; this coincided with morphologically visible changes to the actin cytoskeleton (Figure 6F and Additional file 16). We conclude that actin polymerization is necessary to maintain the morphological integrity of IPMCs.

### Perturbation of actin enhances HIV-1 release from MDMs

**Figure 7:**
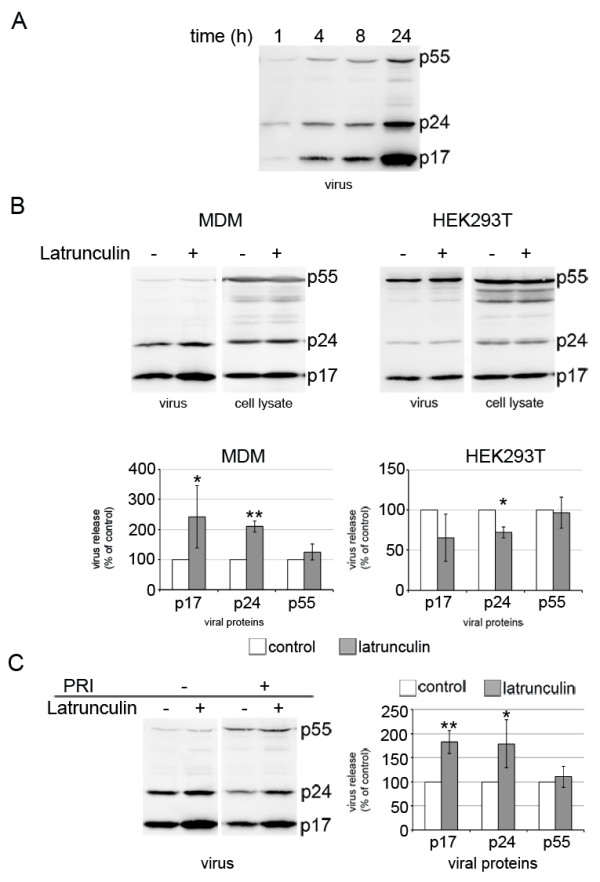
**Perturbation of actin polymerization enhances HIV-1 release from IPMC. (A)** MDMs (4×10^5^) were infected with HIV-1_BaL_ for 7 days. Cells were washed in phosphate-buffered saline, new medium was added and virus released into the culture supernatant was collected at the indicated times, concentrated by centrifugation through a sucrose cushion and subjected to western blotting for HIV-1 Gag proteins. **(B)** MDMs were infected with HIV-1_BaL_ for 7 days, washed and treated with DMSO (control) or 2 μM latrunculin A for 2 hours. Right hand panels: HEK293T cells were transfected with the HIV-1 molecular clone NL4.3-R3A, incubated for 48 hours and treated with DMSO (control) or 2 μM latrunculin A for 2 hours. Viruses released during treatment were concentrated by centrifugation and viral proteins detected by western blotting. The blots were imaged with a LAS4000 CCD camera and quantified using ImageQuant Software, as described in the Methods. Protein band intensities of treated samples were normalized to the DMSO control. The western blots are representative examples of five independent experiments, while the graphs show the average of five independent experiments. **(C)** MDMs infected for 7 days were treated simultaneously with the HIV-1 protease inhibitor amprenavir (PRI) and latrunculin A for 2 hours. The plot on the right shows band intensities of viral proteins quantified using ImageQuant software. The western blot is representative of four independent experiments and the plot shows the average of four independent experiments. Error bars represent standard deviations. Significance is shown as the difference between latrunculin-treated and control cells *P <0.05; **P <0.01.

Although HIV assembly in MDMs occurs primarily in IPMCs [8,9,11,14,15], analysis of the media from HIV-infected MDMs by immunoblotting indicates that HIV-1 can be released from IPMCs (Figure 7A). Given the changes we observed in the appearance of IPMCs when the actin cytoskeleton is disrupted, we asked whether disruption of the actin cytoskeleton would affect HIV-1 release. HIV-infected MDMs were treated with media containing latrunculin or dimethyl sulfoxide (DMSO) for 2 hours, and virus released into the medium during this time was detected by immunoblotting. We observed increased levels of capsid p24 and matrix p17 proteins in media from latrunculin-treated MDMs compared to control cells (Figure 7B). By contrast, HIV-transfected HEK293T cells, which do not develop IPMCs and where virus assembly takes place at the cell surface, did not show significant changes in HIV release over this time period (Figure 7B). Analysis of the media from HIV-infected MDMs by p24-ELISA also revealed enhanced virus release from MDMs treated with latrunculin, cytochalasin D or cytochalasin E (Figure S4D in Additional file 9).

To confirm that latrunculin treatment affects virus release rather than the rate of viral protein synthesis or virus assembly, we inhibited virus maturation in MDMs using the HIV-1 protease inhibitor amprenavir and simultaneously treated the cells with latrunculin. If disruption of the actin cytoskeleton stimulates viral protein synthesis or the rate of assembly, the viruses released in the presence of latrunculin and amprenavir should be mainly immature (that is, contain mostly p55 and not the cleaved p24 and p17 Gag products). By contrast, if HIV assembly and Gag processing happen prior to the latrunculin and amprenavir treatment, the released virus would be mainly mature (that is, low in p55 and high in p24 and p17). We detected the release of p24/p17-containing mature virus particles from control cells after treatment with the HIV-1 protease inhibitor, sugzgesting that this virus had accumulated in IPMCs before the inhibition of virus maturation. In latrunculin- and amprenavir-treated cells, we again detected enhanced virus release, and most of this was in the form of mature virus particles, suggesting that latrunculin induced the release of preformed virus particles from IPMCs (Figure 7C).

Together, these experiments demonstrate that perturbation of the actin cytoskeleton causes the tightly packed IPMC membranes to become more dispersed and may lead to the opening of the narrow channels connecting the compartments to the cell surface, and that this can enhance the release of HIV from the compartments.

## Discussion

For some time, it has been known that in human MDMs, HIV-1 buds into and accumulates in surface-connected intracellular compartments, or IPMCs (also termed virus-containing compartments or VCC [15,27]). Although their origin, organization and function is poorly understood, much of our current knowledge of these compartments derives from EM studies, in which various techniques, including serial sectioning, electron tomography or ion abrasion scanning electron microscopy [10,11,13,15] have indicated that IPMCs consist of complex intracellular networks of membranes, with interconnected vacuole-like and tubular components, and channel-like connections to the cell surface. However, these morphological techniques are limited to the analysis of small portions of the total volume of fixed cells and do not provide information on the dynamics of the compartments in real time. Here we have used fluorescent membrane labels - FM 4–64, CellMask and the PI(4,5)P_2_ probe PH-GFP, in combination with confocal z-series imaging, three-dimensional volume reconstructions and live cell imaging - to study the properties of IPMCs. In agreement with and extending previous studies [10,11,13,15], we show that both uninfected and HIV-1-infected MDMs contain morphologically similar IPMCs that appear as dynamic networks of vacuoles of various sizes, connected to each other and to the cell surface by thinner tubules or closely apposed membrane sheets. Furthermore, we show that the normal morphology of IPMCs is dependent on the integrity of the actin cytoskeleton and that disrupting this integrity can stimulate the release of mature, IPMC-sequestered HIV-1.

EM analysis has previously shown individual HIV-containing vacuoles and/or CD81-, CD9- or CD44-labeled structures within MDMs, suggesting that a single cell may contain several IPMC structures [10,11,13,15]. Our live cell imaging studies, which avoid fixation-induced fragmentation of membrane compartments, indicate that these vacuoles are in most cases sub-domains of single, larger IPMCs. Moreover, although most MDMs contained a single IPMC, three-dimensional reconstruction of complete cells and IPMCs highlighted the complexity and extensive cell-to-cell variability in the size and morphology of IPMCs. The rapid labeling of IPMCs (within minutes), even at 4°C when endocytosis is inhibited, and the visualization of at least one, and frequently more, connections from IPMCs to the cell surface provides additional evidence supporting the notion that the compartment is continuous with the cell surface and accessible to small molecule tracers, as suggested by previous work [11,15,16]. Thus, the term IPMC accurately reflects the fact that these compartments are connected to the cell surface and that the IPMC membrane can be regarded as a sub-domain of the plasma membrane. Although accessible to small tracer molecules, it has been suggested that IPMCs are not accessible to antibodies and that this might protect IPMC-sequestered virus from recognition by neutralizing antibodies [13,28]. However, in our hands at least, IPMCs are accessible to antibodies fed from the cell surface at 37°C [11] and we find that many IPMCs can be accessed by high concentrations of antibodies or the fluid tracer horseradish peroxidase when incubated for 1 hour at 4°C (AP-M, unpublished data).

Significantly, many of the studies described here were performed on uninfected MDMs, demonstrating that IPMCs are not induced by HIV infection, although, as previously shown, the compartment expands in size upon HIV-1 infection [15]. IPMCs are therefore likely to have some as yet unidentified function(s) in uninfected macrophages [11,15]. HIV appears to use pre-existing IPMCs for assembly, suggesting that there is an advantage to the virus to exploit these compartments or that key components required for virus assembly are located within these plasma membrane sub-domains. Currently, it is not clear how HIV targets IPMCs, though the lipid PI(4,5)P_2_, which binds directly to the HIV matrix basic domain and plays a key role in Gag recruitment to the plasma membrane, is likely to be involved [20,29]. We analyzed the distribution of PI(4,5)P_2_ in MDMs, either using the PH-GFP probe or immunostaining with a PI(4,5)P_2_-specific antibody [23,24]. PH-GFP labeled the cell surface as well as IPMCs, indicating that PI(4,5)P_2_ is abundant in these locations. Labeling of IPMCs with anti-PI(4,5)P_2_ antibody required permeabilization with Triton X-100, and was poor after saponin treatment, perhaps indicating the presence of detergent-resistant membranes in IPMCs.

Given that the PH-GFP probe strongly stained IPMC membranes, it could be used to follow the compartment in live cell imaging studies. This allowed, for the first time, studies of the behavior and dynamics of IPMCs in uninfected macrophages. IPMCs labeled with PH-GFP were essentially stable throughout the time of recording, that is, for at least one hour. Similar observations were made with MDMs transfected with Gag-GFP, where IPMCs also appeared stable, though we could occasionally observe changes in the subcellular distribution of Gag-GFP in IPMCs and in IPMC-associated channels. Our studies therefore complement previous analyses with Gag-GFP or biarsenical-tetracysteine-tagged fluorescent Gag [13,30], where accumulations in MDMs were also seen to be comparatively stable. FRAP and FLIP analyses of the PH-GFP probe demonstrated that IPMC membranes are able to rapidly exchange PH-GFP with surrounding membranes and therefore they are dynamic structures. Interestingly, we did not observe any kinetic differences in the behavior of plasma membrane or IPMC-bound PH-GFP in our experiments.

Because IPMCs are coated with actin filaments and, in mature MDMs, the structure of the IPMCs is at least in part stabilized via β2 integrin-containing focal adhesion-like complexes linking to the actin cytoskeleton [14], we investigated the role of actin in the organization of the IPMCs. Inhibitors of actin polymerization (latrunculin, cytochalasin D and cytochalasin E) caused the intracellular accumulations of HIV particles to disperse into smaller pockets of viruses, an effect similar to that seen after depletion of β2 integrins in MDMs [14]. A similar effect was previously described in dendritic cells [31] and may explain the reduction in intracellular Gag accumulation after cytochalasin D treatment of 7-day-old MDMs [12]. Staining with CellMask demonstrated that, although dispersed, the IPMCs remained connected to the plasma membrane in the drug-treated cells. In addition, when MDMs were transfected with PH-GFP and treated with latrunculin, the IPMCs appeared as a meshwork of membranes with clear connections to the cell surface (Additional file 12). We also showed that latrunculin treatment did not inhibit virus assembly in MDMs, but instead enhanced the release of preformed HIV-1 particles, presumably through the dispersed membrane channels. Together these experiments suggest that an intact actin cytoskeleton is required both to maintain the structure of IPMCs and regulate the release of HIV from MDMs. A recent study showed that microtubules also affect the distribution of VCCs in MDMs, and suggested that kinesin family-3A complexes may drive IPMCs toward the plasma membrane and stimulate virus release [27].

## Conclusion

We have demonstrated the complexity and plasticity of IPMCs involved in HIV assembly in MDMs. We show that these structures are resident compartments of MDMs and that in uninfected and HIV-infected cells the integrity of IPMCs is maintained and regulated through the actin cytoskeleton. Although the function of IPMCs in MDMs is unclear, similar intracellular compartments have been seen in dendritic cells, where they are involved in the formation of virological synapses and in the *trans*-infection of T-cells by HIV and perhaps other viruses [31-33]. In this context, remodeling of the actin cytoskeleton may facilitate the recruitment of IPMCs to the sites of cell-cell contact that form virological synapses and may thus be involved in the spread of virus to susceptible target cells.

## Methods

### Reagents and antibodies

Tissue culture media and supplements were purchased from Invitrogen (Paisley, UK), tissue culture plastic from TPP (Trasadingen, Switzerland) and chemicals were from Sigma-Aldrich (Dorset, UK), unless otherwise specified.

Mouse monoclonal antibodies against HIV-1 p24/p55 (38:96K and EF7) and p17 (4C9) were obtained from the National Institute for Biological Standards and Control Center for AIDS Reagents (CFAR, South Mimms, UK). Anti-CD81 (M38) was provided by F. Berditchevski (University of Birmingham, UK); anti-PI(4,5)P_2_, (2C11; IgM) was provided by G. Schiavo (Cancer Research UK, London); anti-CD44 (MEM-85) was from Abcam Ltd. (Cambridge, UK); horseradish peroxidase-conjugated goat anti-mouse IgG(H+L) [F(ab’)_2_] from Thermo Fisher Scientific (Waltham, MA, USA); and Alexa Fluor 594-conjugated phalloidin and Alexa Fluor-labeled antibody reagents were from Invitrogen.

### Preparation of MDMs and infection with HIV-1_BaL_

MDMs, prepared from peripheral blood mononuclear cells isolated from buffy coats from healthy blood donors (National Blood Service, Essex, UK), as described previously [11], were cultured in complete medium (Roswell Park Memorial Institute 1640, 100 U/ml penicillin, 0.1 mg/ml streptomycin, and 10% human AB serum) and differentiated with 10 ng/ml of macrophage colony-stimulating factor (R&D Systems, Abingdon, UK) for 2 days. Unless otherwise specified, the MDMs were used after 14 days in culture. Stocks of HIV-1_BaL_ were prepared as described [11,34]. Where indicated, 7-day-old MDMs were infected with HIV-1_BaL_ (2 FFU/cell) by spinoculation at 2,500 rpm for 2 hours at room temperature, and cultured for a further 7 days.

### Labeling of plasma membrane with lipid dyes

MDMs were washed in ice-cold PBS and incubated with 5 μg/ml FM 4-64FX (Invitrogen) in PBS for 30 minutes on ice, washed in ice-cold PBS and fixed in 4% paraformaldehyde (PFA)/2% glutaraldehyde (TAAB Laboratories, Aldermaston, UK). Alternatively, MDMs were washed in PBS and treated with serum-free MDMs were washed in PBS and treated with serum-free RPMI (Roswell Park Memorial Institute) 1640 containing 2.5 μg/ml CellMask Plasma Membrane Stain (Invitrogen) for 5 minutes at 37°C or for 30 minutes at 4°C, washed in PBS and fixed in 4% PFA.

### Immunofluorescence

MDMs were fixed in 4% PFA, quenched with 50 mM NH_4_Cl and permeabilized with 0.1% Triton X-100 in PBS. After blocking in PBS with 1% BSA, MDMs were labeled for 1 hour with primary antibodies diluted in PBS with 1% BSA, washed, and labeled again with Alexa Fluor-488 or 594-conjugated secondary antibodies for 1 hour. For actin staining, Alexa Fluor 594-conjugated phalloidin was added together with the secondary antibody. Samples were washed in PBS with 1% BSA, mounted in Moviol and analyzed by confocal microscopy. Confocal images were acquired with a Leica SPE confocal microscope, 63× oil objective (NA 1.3) and Leica LAS Software, and processed using Adobe Photoshop, ImageJ and Fiji.

### Immunostaining for PI(4,5)P_2_

In one protocol, MDMs were fixed for 15 minutes in 4% PFA with 2% glutaraldehyde, cooled on ice for 2 minutes and processed at 4°C. Cells were permeabilized for 45 minutes in 0.5% saponin, 5% normal goat serum (NGS), 50 mM NH_4_Cl in Buffer A (20 mM PIPES-NaOH, 137 mM NaCl, 2.7 mM KCl, pH 6.8) and incubated overnight with antibody 2C11 (16 μg/ml, diluted in 0.5% saponin, 5% NGS, 50 mM NH_4_Cl in Buffer A), washed in buffer A, and stained with Alexa Fluor 488-labeled goat anti-mouse IgM for 1 hour. Cells were washed in buffer A and fixed in ice-cold 2% PFA for 15 minutes before mounting and imaging.

In a second staining protocol, MDMs were fixed in 4% PFA for 15 minutes at room temperature, cooled on ice for 2 minutes and permeabilized in 0.2% Triton X-100, 5% NGS in PBS for 45 minutes at 4°C. Cells were incubated with 2C11 (diluted in 0.2% Triton X-100, 5% NGS in PBS) overnight at 4°C, washed in PBS and stained with a second antibody as above.

### Plasmids and nucleofection

The PH domain of phospholipase Cδ cloned into pEGFP-N1 (PH-GFP) was obtained from S. Cockroft (University College London, UK) and the 2xFYVE domain cloned into pEGFP-N1 from H. Stenmark (The Norwegian Radium Hospital, Oslo, Norway). LifeAct-Ruby was obtained from D. Cutler (Medical Research Council Laboratory for Molecular Cell Biology, University College London, London, UK). Codon-optimized Gag-GFP cloned into pEGFP-N1 was from W. Sundquist (University of Utah School of Medicine, Salt Lake City, UT, USA). MDMs were nucleofected with 5 μg of plasmid DNA using the macrophage nucleofection kit (Amaxa Biosystems, Cologne, Germany) according to the manufacturer’s instructions, seeded into four-well Lab-Tek Chambered Coverglass units (Thermo Fisher Scientific) and incubated at 37°C for 24 hours.

### Live cell microscopy

MDMs nucleofected as above were imaged with an UltraVIEW Vox spinning disc confocal system (PerkinElmer, Cambridge, UK) fitted on a Nikon ECLIPSE Ti microscope equipped with a temperature and CO_2_-controllable environment chamber. All images were taken with a Nikon 60× NA 1.2 oil objective. Volocity 5.3.2 (Perkin Elmer) was used for image acquisition and figures were constructed using Volocity, Adobe Photoshop, ImageJ and Fiji software.

### FRAP and FLIP analysis

FRAP measurements were carried out at 37°C using an UltraVIEW Vox spinning disc microscope with a Nikon 100× NA 1.4 oil objective. After acquiring the base level of fluorescence for 2 seconds, the region of interest (IPMC in MDMs) was photobleached using a 20 millisecond laser pulse (488 nm laser power at 50% intensity), and fluorescence recovery was monitored for 20 seconds by collecting frames at maximum speed. For FLIP analysis, a region of interest was bleached 10 times, consecutively, using the 488 nm laser at 50% intensity and the UltraVIEW PK device. After each bleaching pulse, fluorescence recovery was monitored in the bleached region and at other areas of interest over 10 seconds. Data were analyzed and the images processed as above.

### Electron microscopy

HIV-infected MDMs were fixed and embedded in Epon or prepared for cryosectioning and immunolabeling as described previously [11,14]. Ultrathin sections were inspected with a Tecnai G2 Spirit transmission EM (FEI, Eindhoven, The Netherlands) and digital images recorded with a Morada 11 MegaPixel TEM camera (Olympus Soft Imaging Solutions, Münster, Germany) and AnalySIS software. Images were adjusted for brightness and contrast, and figures were assembled with Adobe Photoshop.

### Virus release assay and western blot analysis

HIV-infected MDMs were washed twice with PBS and incubated for 2 hours in medium containing either 2 μM latrunculin A (Invitrogen), 5 μM cytochalasin D (Invitrogen), or 1 μM cytochalasin E (MP Biomedicals, Solon, OH, USA) or carrier (DMSO). HEK293T cells were transfected with the HIV-1 molecular clone NL4.3-R3A [35] and incubated for 48 hours at 37°C. The cells were treated with 2 μM latrunculin A or DMSO for 2 hours and virus-containing media were collected. Cell debris was removed by centrifugation (2,000 rpm, 5 minutes) and the viruses concentrated by centrifugation (47,000 rpm, 2 hours) through a 20% sucrose cushion. Cells and virus pellets were lysed in Laemmli SDS sample buffer at 96°C for 10 minutes and the proteins separated on 12% polyacrylamide gels. Subsequently, the proteins were transferred onto polyvinylidene difluoride membranes (Millipore, Billerica, MA, USA); the membranes were quenched, and specific proteins detected using antibodies to p24/p55 or p17 and horseradish peroxidase-conjugated goat anti-mouse secondary antibodies. Labeled protein bands were detected using SuperSignal West Dura Substrate (Thermo Fisher Scientific) and band intensities were recorded and quantified using a LAS4000 CCD camera and ImageQuant software (GE Healthcare, Buckinghamshire, UK).

## Abbreviations

BSA: Bovine serum albumin; DMSO: Dimethyl sulfoxide; ELISA: Enzyme-linked immunosorbent assay; EM: Electron microscopy; FLIP: Fluorescence loss in photobleaching; FRAP: Fluorescence recovery after photobleaching; Gag-GFP: HIV Gag p55 linked to GFP; GFP: Green fluorescent protein; Ig: Immunoglobulin; IPMC: Intracellular plasma membrane-connected compartment; MDM: Monocyte-derived macrophage; NGS: Normal goat serum; PBS: Phosphate-buffered saline; PFA: Paraformaldehyde; PH: Pleckstrin homology; PH-GFP: Phospholipase Cδ pleckstrin homology domain linked to GFP; PI(4,5)P2: Phosphatidylinositol 4,5-bisphosphate.

## Competing interests

The authors declare that they have no competing interests.

## Authors’ contributions

PM, AP-M and MM designed the experiments. PM and AP-M performed the experiments. PM, AP-M and MM analyzed the data, and wrote the manuscript. All authors read and approved the final manuscript.

## Supplementary Material

Additional file 1: Movie 1 3D reconstruction of an uninfected MDM labeled with FM 4-64FX. Uninfected MDMs were labeled with 5 μg/ml FM 4-64FX, fixed and analyzed by confocal microscopy. Fiji software was used to assemble a 3D reconstruction from 131 optical z-slices (step size of 0.04 μm). Cell as shown in Figure 1A-C. Click here for file

Additional file 2: Figure S1 Cell-to-cell variability in the size and morphology of the IPMCs. (A) MDMs were nucleofected to express Gag-GFP and stained with FM 4-64FX 24 hours later. Confocal sections show co-localization of Gag-GFP and the FM 4–64 dye in the IPMC. (B) MDMs were labeled with the membrane-impermeable CellMask for 5 minutes at 37°C (or 30 minutes at 4°C; bottom row in panel B) and fixed. Confocal sections show labeled cell surface and IPMCs in uninfected and HIV-infected MDMs from the same donor. (C) MDMs were nucleofected with PH-GFP. Confocal sections show labeled cell surface and IPMCs in uninfected and HIV-infected MDMs from the same donor*.* Infected MDMs were detected by staining for the HIV matrix protein p17 (bottom panels). All scale bars: 10 μm. Click here for file

Additional file 3: Movie 2 3D reconstruction of an uninfected MDM labeled with CellMask. Uninfected MDMs were labeled with CellMask for 5 minutes at 37°C. Confocal sections were recorded using an UltraVIEW Vox spinning disc confocal system (PerkinElmer, Cambridge, UK). Fiji software was used to build this 3D reconstruction assembled from 165 optical z-slices (step size of 0.1 μm). Cell as shown in Figure 1D-F. Click here for file

Additional file 4: Movie 3 3D reconstruction of an uninfected MDM expressing PH-GFP. Uninfected MDMs were nucleofected to express PH-GFP for 24 hours, fixed and imaged by confocal microscopy. Fiji software was used to assemble a 3D reconstruction from 230 optical z-slices (step size of 0.04 μm). Cell as shown in Figure 1I-K. Click here for file

Additional file 5: Figure S2 Immunostaining for PI(4,5)P_2_ in MDMs. MDMs were either (A) fixed with 4% paraformaldehyde/2% glutaraldehyde and permeabilized with 0.5% saponin or (B) fixed with 4% paraformaldehyde and permeabilized in 0.2% Triton X-100. Cells were labeled with a mouse monoclonal anti-PI(4,5)P_2_ antibody 2C11 and co-stained for CD81. Scale bars: 10 μm. Click here for file

Additional file 6: Movie 4 Live cell imaging of an uninfected MDM nucleofected with PH-GFP. MDMs were nucleofected with PH-GFP and imaged after 24 hours using an UltraVIEW Vox spinning disc confocal system fitted on a Nikon ECLIPSE Ti microscope equipped with a temperature and CO_2_-controllable environment chamber. The movie was assembled from images taken every 10 seconds. Cell as shown in Figure 3A. Click here for file

Additional file 7: Movie 5 Live cell imaging of an uninfected MDM expressing PH-GFP. MDMs were nucleofected to express PH-GFP and imaged after 24 hours using an UltraVIEW Vox spinning disc confocal system fitted on a Nikon ECLIPSE Ti microscope equipped with a temperature and CO_2_-controllable environment chamber. The movie was assembled from images taken every 10 seconds. Cell as shown in Figure 3B. Click here for file

Additional file 8: Figure S3 Latrunculin A induces the translocation of actin into nuclei. MDMs were treated with 2 μM latrunculin A or DMSO (control) for 2 hours. Cells were stained with Alexa Fluor 594-conjugated phalloidin to label actin and 4′,6-diamidino-2-phenylindole to label nuclei. The images show single optical sections acquired with a Leica SPE confocal microscope. Scale bars: 10 μm. Click here for file

Additional file 9: Figure S4 Latrunculin A, cytochalasin E or cytochalasin D alter IPMC morphology and enhance HIV-1 release from MDMs. HIV-infected MDMs were treated with 2 μM latrunculin A (Lat), 1 μM cytochalasin E (CCE), 5 μM cytochalasin D (CCD) or DMSO (control) for 2 hours. (A) Cells were stained with an anti-p17 antibody that only recognizes mature virus particles and Alexa Fluor 594-conjugated phalloidin to label actin. The images show single optical sections acquired with a Leica SPE confocal microscope. The cells marked by white squares are enlarged in the bottom row. Scale bars: 10 μm. (B) Single optical sections showing examples of compact, dispersed or both (mixed) compartments. Cells were stained with antibodies against CD81 and p17. (C) MDMs were analyzed according to the morphology of the IPMCs. Ten single optical sections through the cells were acquired, inspected for the presence of IPMCs, and cells containing either compact or dispersed IPMCs or both (mixed) were counted. (D) The amount of virus released during treatment of MDMs with the actin polymerization inhibitors was analyzed by p24 ELISA assay (AIDS and Cancer Virus Program NCI-Frederick, MD, USA). Results are shown relative to the control untreated MDMs (DMSO). Click here for file

Additional file 10: Figure S5 HIV particles still assemble in IPMCs after treatment with latrunculin A. HIV-infected MDMs were treated with DMSO or 2 μM Latrunculin A for 2 hours and processed for cryosectioning. Ultrathin cryosections from (A, B) infected control or (C, D, E) latrunculin A-treated macrophages were immunolabeled with anti-p24 antibodies, a rabbit anti-mouse bridging antibody and protein A-gold (5 nm in A, B and D, or 10 nm in C and E). EM analysis showed that HIV particles accumulated in complex IPMCs in both control and latrunculin A-treated cells. Arrows in B and D show representative immature virus particles and budding profiles, indicating virus assembly within IPMCs. Arrowheads in E mark an example of the electron-dense coats containing β2-integrins and focal adhesion proteins [14]. Images were taken on an FEI Tecnai G2 Spirit transmission EM with a Morada 11 MegaPixel TEM camera and AnalySIS software. Scale bars: 1 μm in A and C, and 200 nm in B, D, E. Click here for file

Additional file 11: Movie 6 3D reconstruction of an infected MDM labeled with CellMask and treated with latrunculin A. MDMs infected for 7 days with HIV-1_BaL_ were treated with DMSO (control) or latrunculin A for 2 hours and labeled with CellMask. Fiji software was used to build a 3D reconstruction of an IPMC from 200 optical *z-*slices (step size of 0.04 μm). The 3D reconstructions were cut out from whole cells to display the individual compartments. The control cell is shown on the left, and the reconstruction from a latrunculin-treated cell on the right. Note multiple connections to the cell surface. Click here for file

Additional file 12: Figure S6 Effect of latrunculin A on PH-GFP expressing MDMs. MDMs were nucleofected to express PH-GFP for 24 hours and then treated with DMSO (control) or latrunculin A for 2 hours. (A) The images show single optical sections acquired with a Leica SPE confocal microscope. (B) Both 3D reconstructions of IPMCs were built from 142 optical z-slices (step size of 0.04 μm). The 3D reconstructions were cut out from whole cells to display the individual compartments. Scale bars: 10 μm. Click here for file

Additional file 13: Movie 7 3D reconstruction of uninfected MDMs expressing PH-GFP and treated with latrunculin A. MDMs were nucleofected to express PH-GFP for 24 hours and then treated with DMSO (control) or latrunculin A for 2 hours. Fiji software was used to build 3D reconstructions of IPMCs from 142 optical z-slices (step size of 0.04 μm). The 3D reconstructions were cut out from the whole cell to better display the individual compartments. The control cell is shown on the left, and the reconstruction from a latrunculin-treated cell on the right. Note multiple connections to the cell surface. Cells as shown in Figure S6 in Additional file 12. Click here for file

Additional file 14: Movie 8 Live cell imaging of MDMs expressing Gag-GFP. Cells were imaged with the UltraVIEW Vox spinning disc confocal system. Images were recorded at 6 frames/minute. Cell as shown in Figure 6D. Click here for file

Additional file 15: Movie 9 Live cell imaging of Gag-GFP in an IPMC. MDMs were nucleofected to express Gag-GFP and imaged with the UltraVIEW Vox spinning disc confocal system. Images were recorded at 6 frames/minute. Cell as shown in Figure 6E. Click here for file

Additional file 16: Movie 10 Live cell imaging of MDMs expressing Gag-GFP and LifeAct-Ruby: Effect of latrunculin treatment. MDMs were nucleofected to express Gag-GFP and LifeAct-Ruby for 24 hours then treated with 2 μM latrunculin A and imaged for 1.5 hours with the UltraVIEW Vox spinning disc confocal system. Images were recorded at 6 frames/minute. Cell as shown in Figure 6F. Click here for file
