# Supplementary figures and images for: Organization and regulation of intracellular plasma membrane-connected HIV-1 assembly compartments in macrophages
Source: BMC Biol. 2013 Aug 2;11:89. doi: 10.1186/1741-7007-11-89 (PMC3751737; doi:10.1186/1741-7007-11-89)

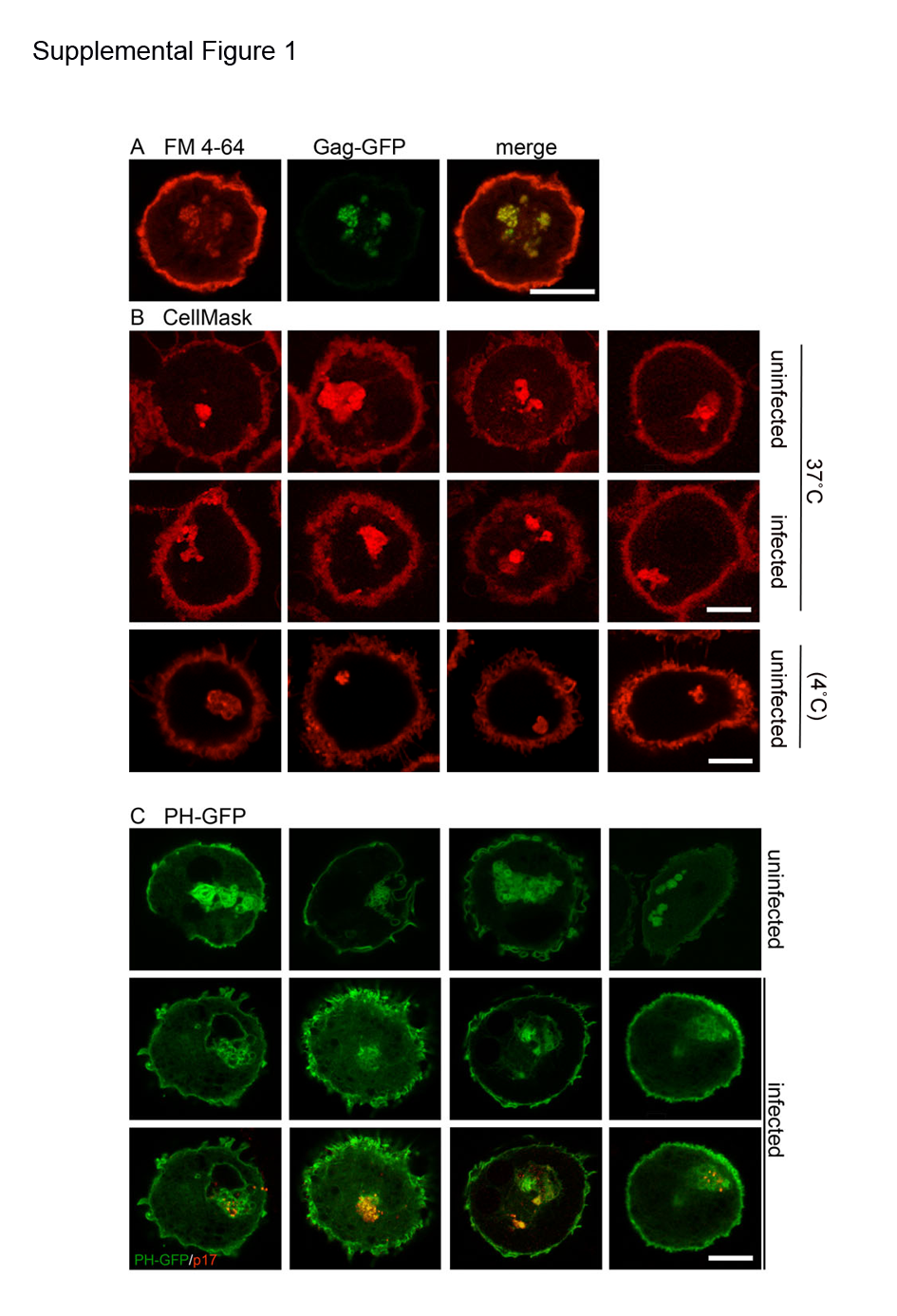

Supplement: Additional file 2: Figure S1 — Cell-to-cell variability in the size and morphology of the IPMCs. (A) MDMs were nucleofected to express Gag-GFP and stained with FM 4-64FX 24 hours later. Confocal sections show co-localization of Gag-GFP and the FM 4–64 dye in the IPMC. (B) MDMs were labeled with the membrane-impermeable CellMask for 5 minutes at 37°C (or 30 minutes at 4°C; bottom row in panel B) and fixed. Confocal sections show labeled cell surface and IPMCs in uninfected and HIV-infected MDMs from the same donor. (C) MDMs were nucleofected with PH-GFP. Confocal sections show labeled cell surface and IPMCs in uninfected and HIV-infected MDMs from the same donor. Infected MDMs were detected by staining for the HIV matrix protein p17 (bottom panels). All scale bars: 10 μm. [file 1741-7007-11-89-S2.tiff]

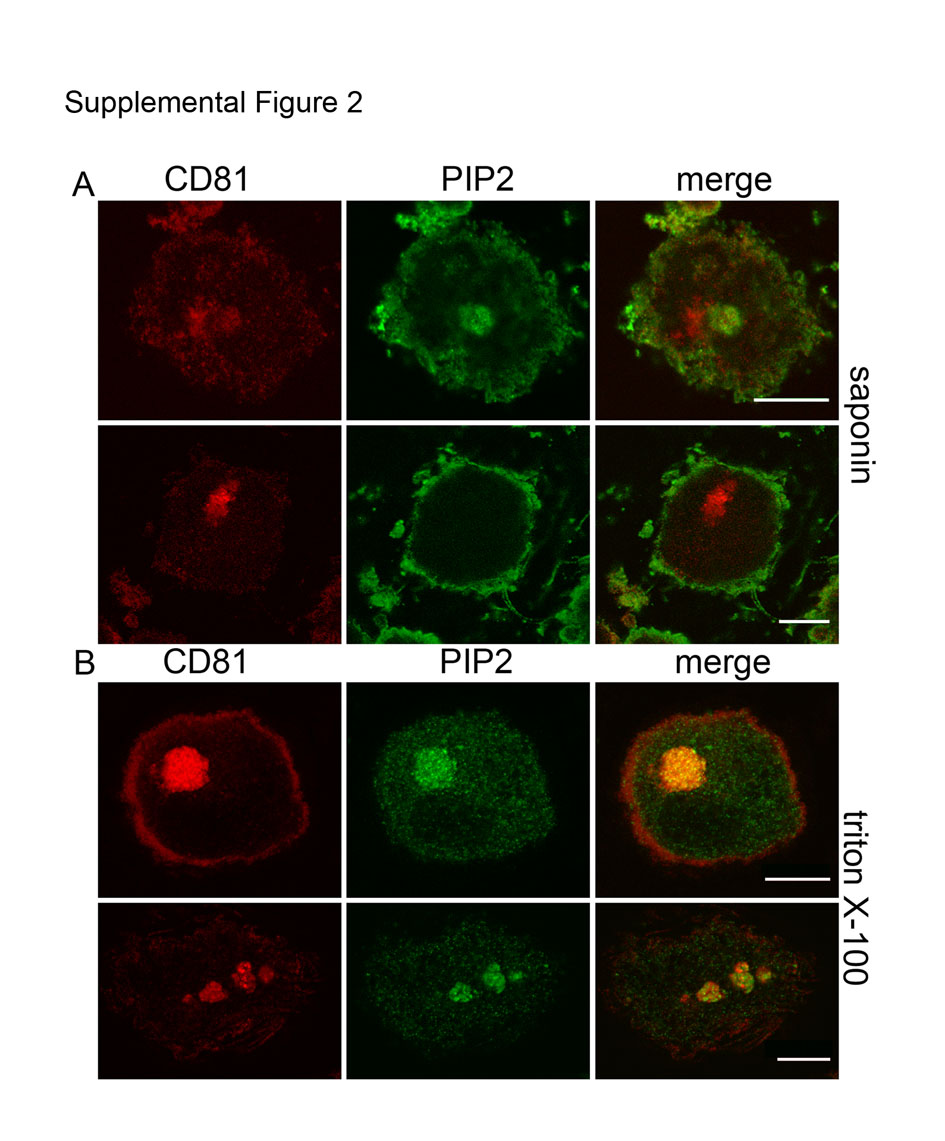

Supplement: Additional file 5: Figure S2 — Immunostaining for PI(4,5)P2 in MDMs. MDMs were either (A) fixed with 4% paraformaldehyde/2% glutaraldehyde and permeabilized with 0.5% saponin or (B) fixed with 4% paraformaldehyde and permeabilized in 0.2% Triton X-100. Cells were labeled with a mouse monoclonal anti-PI(4,5)P2 antibody 2C11 and co-stained for CD81. Scale bars: 10 μm. [file 1741-7007-11-89-S5.tiff]

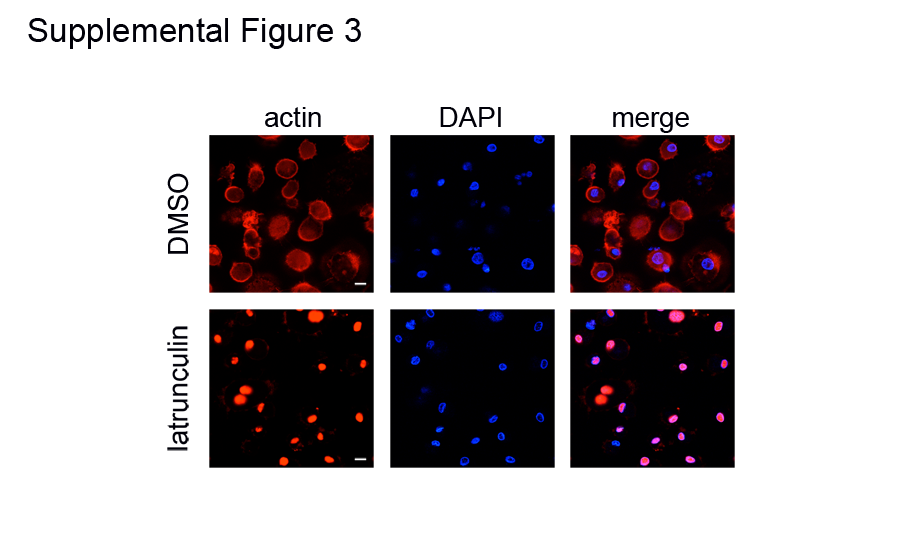

Supplement: Additional file 8: Figure S3 — Latrunculin A induces the translocation of actin into nuclei. MDMs were treated with 2 μM latrunculin A or DMSO (control) for 2 hours. Cells were stained with Alexa Fluor 594-conjugated phalloidin to label actin and 4′,6-diamidino-2-phenylindole to label nuclei. The images show single optical sections acquired with a Leica SPE confocal microscope. Scale bars: 10 μm. [file 1741-7007-11-89-S8.tiff]

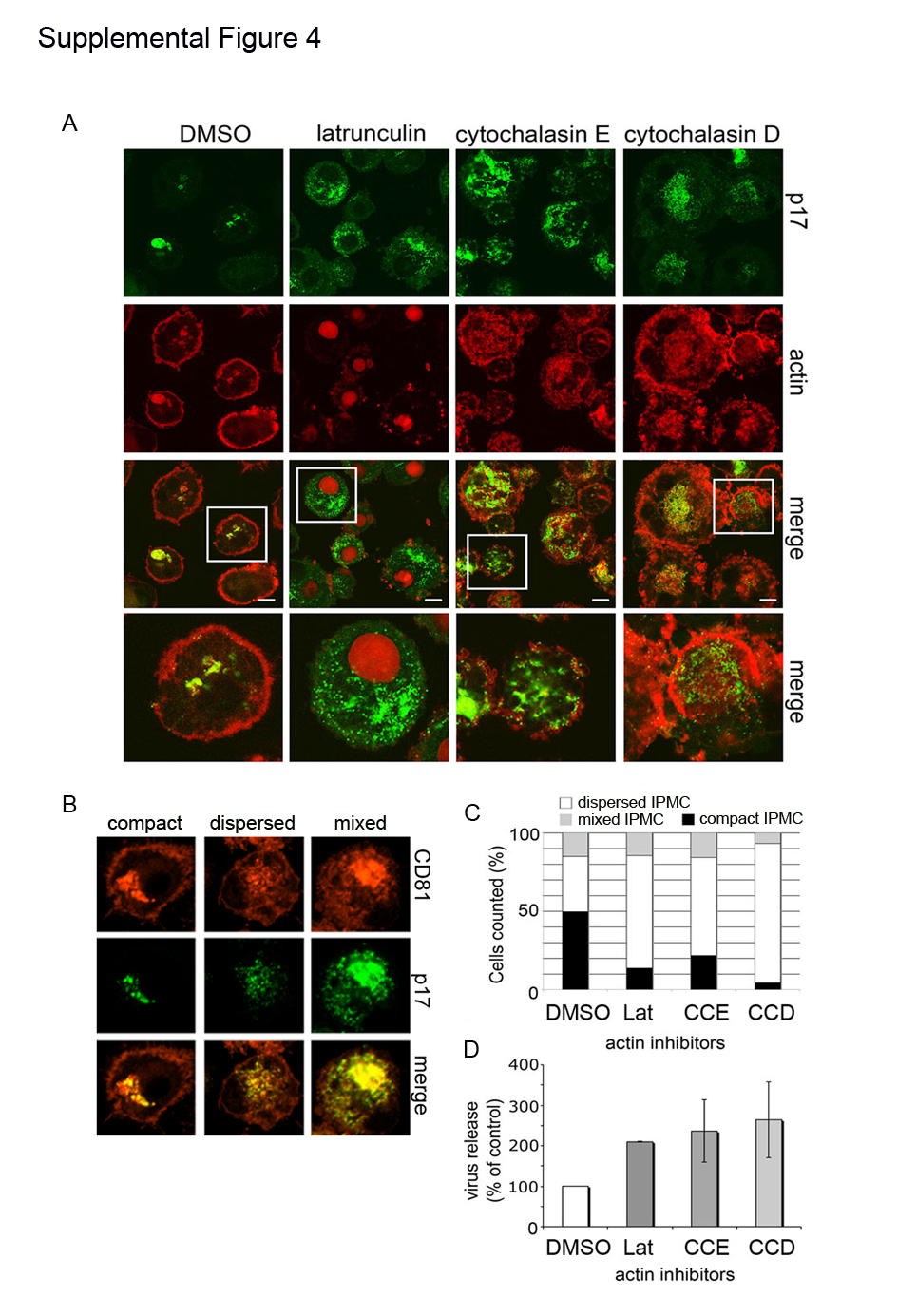

Supplement: Additional file 9: Figure S4 — Latrunculin A, cytochalasin E or cytochalasin D alter IPMC morphology and enhance HIV-1 release from MDMs. HIV-infected MDMs were treated with 2 μM latrunculin A (Lat), 1 μM cytochalasin E (CCE), 5 μM cytochalasin D (CCD) or DMSO (control) for 2 hours. (A) Cells were stained with an anti-p17 antibody that only recognizes mature virus particles and Alexa Fluor 594-conjugated phalloidin to label actin. The images show single optical sections acquired with a Leica SPE confocal microscope. The cells marked by white squares are enlarged in the bottom row. Scale bars: 10 μm. (B) Single optical sections showing examples of compact, dispersed or both (mixed) compartments. Cells were stained with antibodies against CD81 and p17. (C) MDMs were analyzed according to the morphology of the IPMCs. Ten single optical sections through the cells were acquired, inspected for the presence of IPMCs, and cells containing either compact or dispersed IPMCs or both (mixed) were counted. (D) The amount of virus released during treatment of MDMs with the actin polymerization inhibitors was analyzed by p24 ELISA assay (AIDS and Cancer Virus Program NCI-Frederick, MD, USA). Results are shown relative to the control untreated MDMs (DMSO). [file 1741-7007-11-89-S9.tiff]

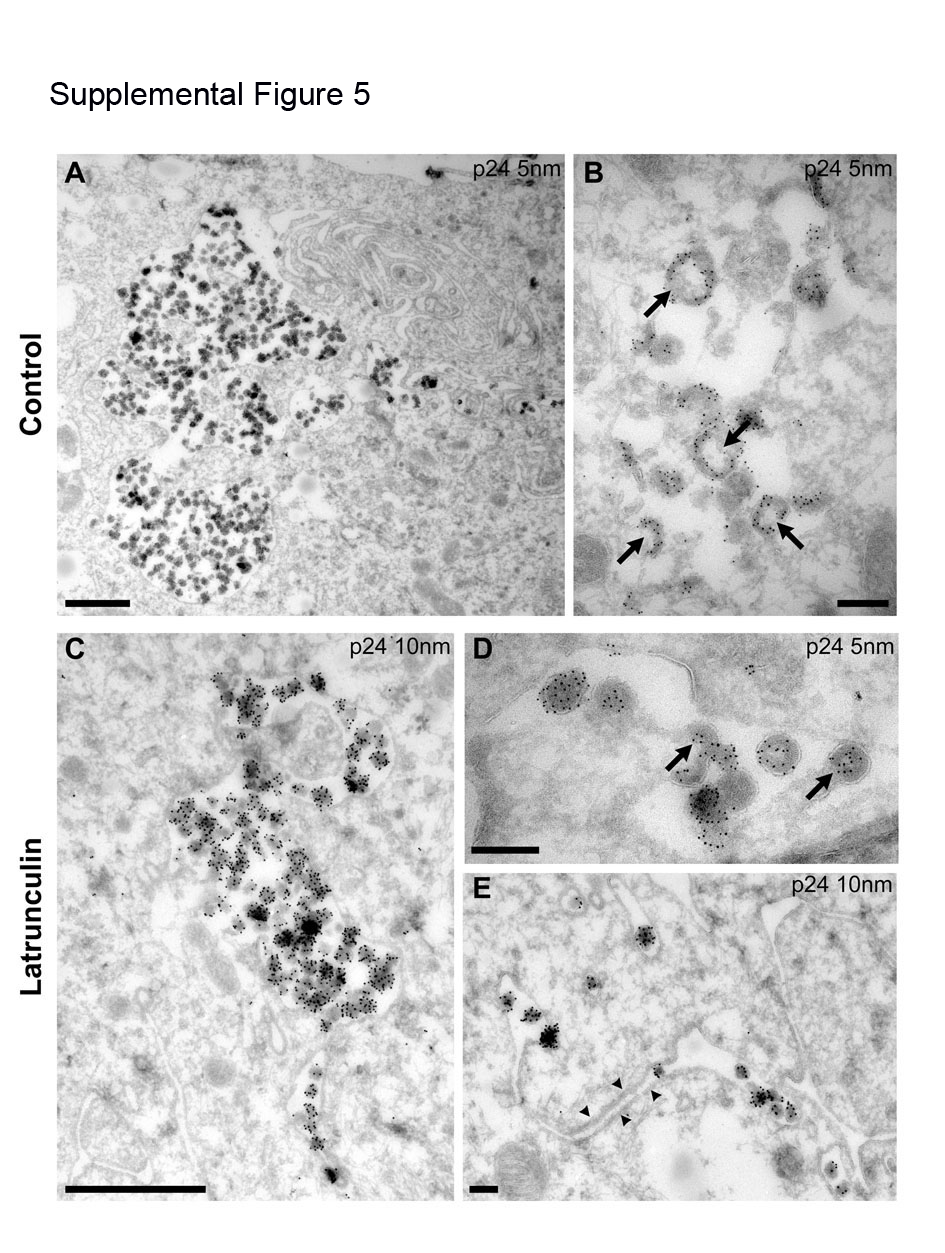

Supplement: Additional file 10: Figure S5 — HIV particles still assemble in IPMCs after treatment with latrunculin A. HIV-infected MDMs were treated with DMSO or 2 μM Latrunculin A for 2 hours and processed for cryosectioning. Ultrathin cryosections from (A, B) infected control or (C, D, E) latrunculin A-treated macrophages were immunolabeled with anti-p24 antibodies, a rabbit anti-mouse bridging antibody and protein A-gold (5 nm in A, B and D, or 10 nm in C and E). EM analysis showed that HIV particles accumulated in complex IPMCs in both control and latrunculin A-treated cells. Arrows in B and D show representative immature virus particles and budding profiles, indicating virus assembly within IPMCs. Arrowheads in E mark an example of the electron-dense coats containing β2-integrins and focal adhesion proteins [14]. Images were taken on an FEI Tecnai G2 Spirit transmission EM with a Morada 11 MegaPixel TEM camera and AnalySIS software. Scale bars: 1 μm in A and C, and 200 nm in B, D, E. [file 1741-7007-11-89-S10.tiff]

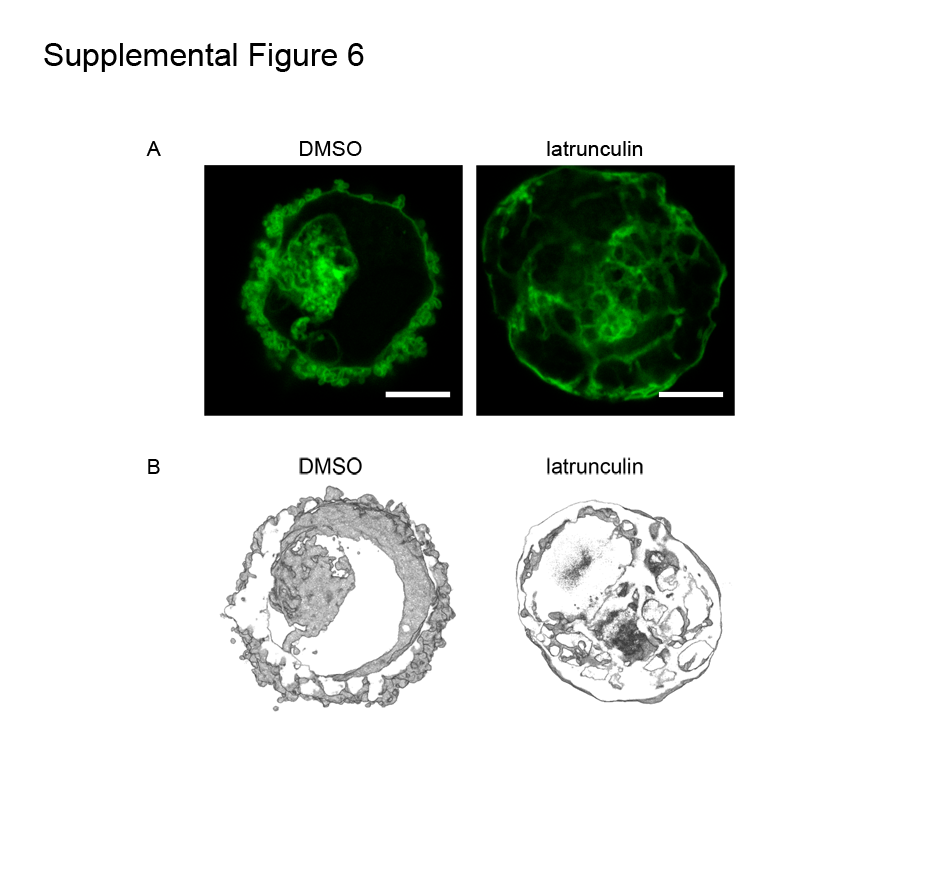

Supplement: Additional file 12: Figure S6 — Effect of latrunculin A on PH-GFP expressing MDMs. MDMs were nucleofected to express PH-GFP for 24 hours and then treated with DMSO (control) or latrunculin A for 2 hours. (A) The images show single optical sections acquired with a Leica SPE confocal microscope. (B) Both 3D reconstructions of IPMCs were built from 142 optical z-slices (step size of 0.04 μm). The 3D reconstructions were cut out from whole cells to display the individual compartments. Scale bars: 10 μm. [file 1741-7007-11-89-S12.tiff]
